# Co-ordination of cell cycle, migration and stem cell-like activity in breast cancer

**DOI:** 10.18632/oncotarget.2312

**Published:** 2014-08-04

**Authors:** Rebecca Lamb, Michael P. Lisanti, Robert B. Clarke, Göran Landberg

**Affiliations:** ^1^ Breakthrough Breast Cancer Unit, Institute of Cancer Sciences, Paterson Institute for Cancer Research, University of Manchester, Wilmslow Road, Manchester, UK; ^2^ Center for Molecular Pathology, Department of Laboratory Medicine, Lund university, Skåne University Hospital, Malmö, Sweden; ^3^ Sahlgrenska Cancer Center, University of Gothenburg, Sweden

**Keywords:** Breast Cancer, Cellular proliferation, Cell migration, Cancer Stem cells

## Abstract

Migration, proliferation and stem cell-like activity are all key cellular characteristics which aid the formation and progression of breast cancer, in addition to involvement in treatment resistance. Many current therapies aim to target tumour proliferation, and although successful, mortality rates in breast cancer remain significant. Our main objectives were to investigate the relationship between proliferation, migration and stem cell-like activity in breast cancer.

We used a panel of cell lines and primary human breast cancer samples to assess the relationship between migration, proliferation and stem cells. We performed live cell sorting according to cell cycle (Hoechst-33324) and in combination with stem-cell markers (CD44/CD24/ESA) followed by assessment of migration and stem cell activity (mammosphere formation).

We identified an inverse relationship between proliferation and migration/stem cell-like activity. G0/1 cells showed increased migration and mammosphere formation. Furthermore we identified a subpopulation of low proliferative stem-like cells (CD44+/24lo/ESA+) with increased migration and mammosphere formation that are specifically inhibited by Dickkopf 1 (DKK1) and Dibenzazepine (DBZ) known stem-cell inhibitors.

These data show the co-ordination of migration, proliferation and stem cell activity in breast cancer, and has identified a sub-population of stem-like cells, greatly adding to our understanding of the complex nature of stem cell biology.

## INTRODUCTION

Breast cancer is one of the most common diseases in women in the Western world, but despite the introduction of anti-cancer treatments such as radiotherapy and targeted drugs such as the anti-oestrogen Tamoxifen, a significant proportion of patients are either resistant to treatment or show disease recurrence. Given that breast cancer currently accounts for approximately 200 000 deaths each year and that the incidence of breast cancer is increasing worldwide, it is essential that we have a better understating of tumour characteristics in order to develop more effective targeted therapies [1-3].

Recurrences at metastatic sites, in particular lung and bone represent the major cause of mortality in breast cancer patients [4, 5]. Migration is a key cellular feature for many cancers including breast cancer thought to be essential in the metastatic process. Tumour cells must possess the ability to migrate and invade into the surrounding tissue in order to leave the primary tumour site. Cells that possess this ability are then able to enter the blood stream and lymphatic system, followed by subsequent colonization of surrounding tissue and formation of metastasis [6]. A number of genes that regulated migration have been identified in many cancers including breast cancer with the most characterised being E-cadherin, a protein which maintains cell-cell adhesion. Down regulation of E-cadherin in breast cancer is well documented and leads to increased migration [7].

A number of general tumour characteristics have been described with loss of control of proliferation considered a hallmark of many cancer types including breast cancer. Normal cellular proliferation is a highly regulated process however when the signals that control proliferation are deregulated, cancer may develop. This deregulation of proliferation may occur due to epithelial mutations or altered regulation of genes that control growth and proliferation, with numerous tumour suppressor genes having been identified. Furthermore, surrounding cells within the tumour stroma may secrete growth factors which in turn allow the uncontrolled proliferation of the cancer cell [8].

Stem cells or cells that possess stem-like cell properties are also thought to be essential in breast cancer initiation and progression. Tumours are heterogeneous in nature and contain a small pool of cells, “cancer stem cells” (CSC), which are suggested to be responsible for regeneration of tumours [9]. CSCs may be identified by cellular markers CD44^+^/24^−^, or by mammosphere formation and self-renewal [10, 11]. Furthermore, cells that possess stem cell-like properties are thought to evade current therapies usually designed to reduce tumour cell proliferation, and have been implicated in treatment resistance, emphasizing the need for finding new treatment strategies [11-13].

Given the importance of migration, proliferation, and stem cell activity, and in particular the role of stem cells in treatment resistance we aimed to investigate the relationship between these key cellular characteristics in breast cancer cell lines and primary human breast cancer samples for validation. Using live cell sorting we have demonstrated a clear inverse relationship between proliferation and migration and stem cell-like activity, with cells within G0/1 stage of the cell cycle having increased migration and mammosphere formation. Furthermore, using the currently defined cell surface markers of breast cancer stem cells (CD44+/24-) we have identified enrichment of stem cell-like activity and migration within low proliferative cells, and showed differential effects of stem cell signalling inhibitors (DKK1 and DBZ) within subgroups of stem-like cells dependant upon their proliferative status. These data add significantly to our understanding of the complex co-coordination of key cellular characteristics in breast cancer and add further to our understanding of stem cells in breast cancer.

## RESULTS

### G0/1 cells breast cancer cells show increased mammosphere formation and migration

**Figure 1:**
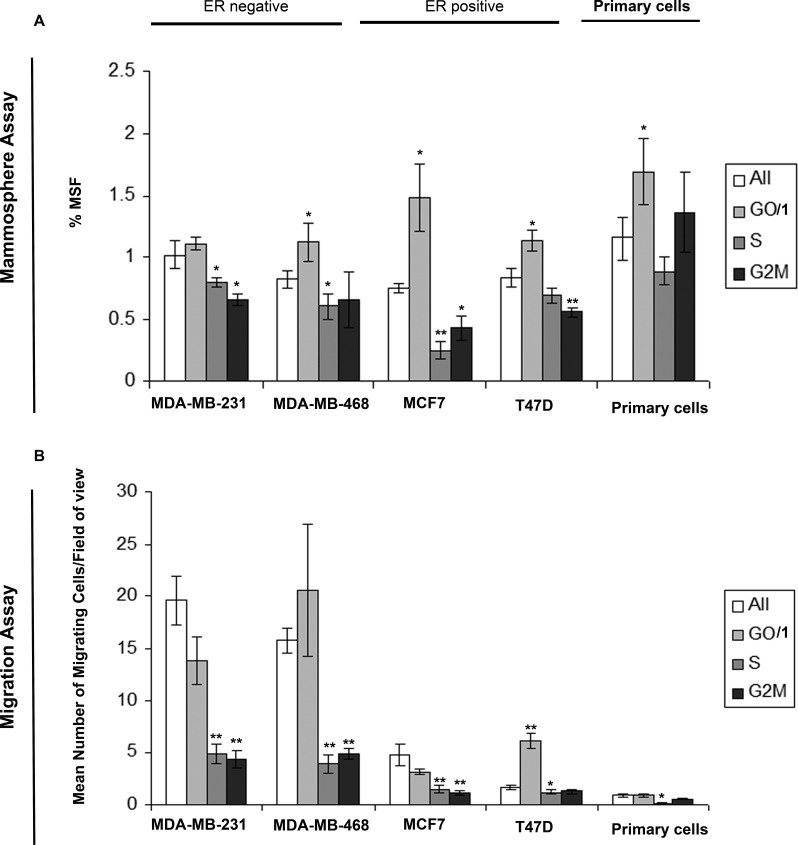
Cells within G0/1 cell cycle phase show increased mammosphere formation and migration Breast cell lines (MDA-MB-231, MDA-MB-468, MCF7 and T47D) and primary human breast cancer cells were live cell sorted by FACS according to DNA profiles (Hoechst staining) into G0/1, S, G2/M and for comparison the whole cell population (all). (A) mammosphere formation and (B) transwell migration assayed. Bar charts represent the mean number of migrated cells/field of view and % mammosphere formation, ±SEM. p values were generated using a two sided t-test assuming equal variance. * indicates significance, p<0.05.

We evaluated the migratory capabilities and mammosphere activity, a known marker of stem-like cells, within differing cell cycles of breast cancer. We generated DNA profiles by Hoechst labelling, and sorted the cells into G0/1, S, G2/M cell cycles phases and for comparison the whole cell population. Experiments were carried out in two ER-ve breast cancer cell lines (MDA-MB-231 and MDA-MB-468), two ER+ve cell lines (MCF7 and T47D) and three primary human breast cancer samples. Cells within the G0/1, and therefore non-proliferative state showed increased mammosphere formation (Fig. 1A) and increased migration (Fig. 1B) compared to cells within S or G2/M (proliferative). Additional labelling with Pyronin Y allowed the separation of G0 and G1 cell populations. We observed no significant difference in either migration or mammosphere formation between G0 and G1 cell cycle phases (Supplementary figure 1). These data demonstrate the inverse link between proliferation, and migration/stem-like cell activity in breast cancer. Furthermore the data provide support for the existence of a non-proliferative stem cell.

### G0/1 Stem-like cells have increased mammosphere activity

**Figure 2:**
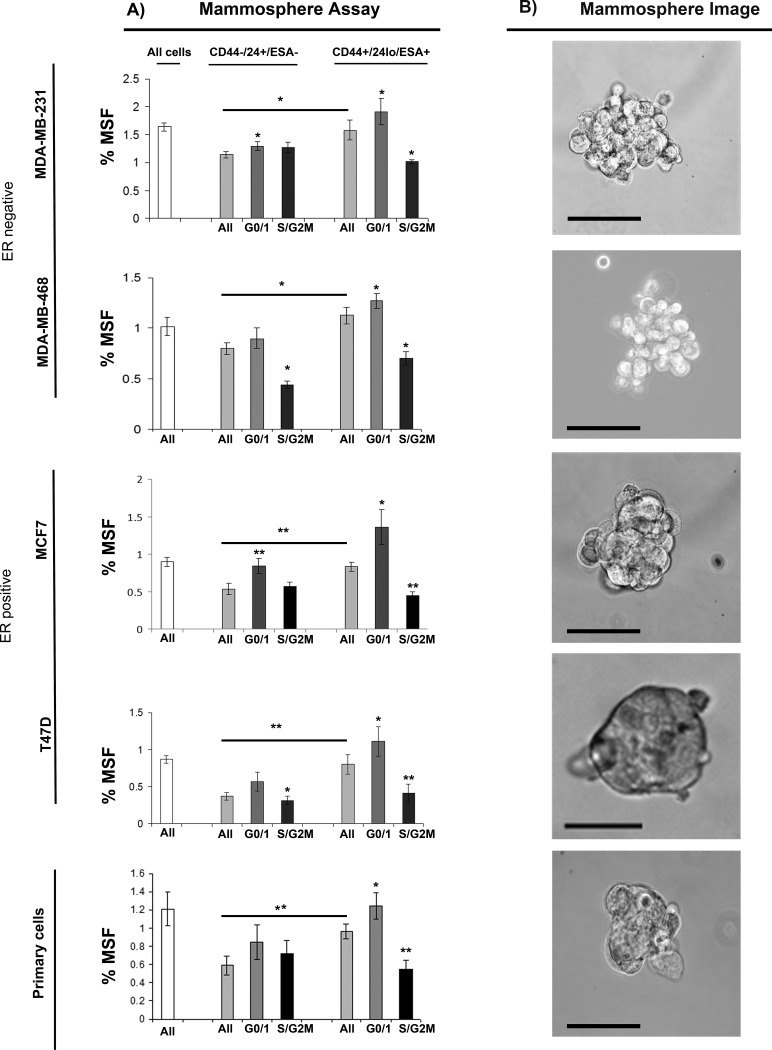
G0/1 Stem-like cells have increased mammosphere activity Breast cancer cell lines (MDA-MB-231, MDA-MB-468, MCF7 and T47D) and primary human breast cancer cells were live cell sorted by FACS. Cells were sorted by ESA +/−, then CD44+/−, then CD24 lo/high to gain CD44+/24lo/ESA+ (Stem-like cells) and CD44-/24hi/ESA- (Non stem-like cells). These populations were sorted according to cell cycle (Hoechst DNA profile) into G0/1 and S/G2/M, and for comparison the whole cell population (all). (A) mammosphere formation assayed. Bar charts represent the % mammosphere formation, ±SEM. p values were generated using a two sided t-test assuming equal variance. * indicates significance, p<0.05 B) Representative images of mammospheres. Scale bar represent 50μm.

We next evaluated further the relationship between cell cycle and stem-like cells using multi-colour live FACS cell sorting. (Fig. 2A) Breast cancer cell lines (MDA-MB-231, MDA-MB-468, MCF7 and T47D) and primary human breast cancer cells were labelled with pre-conjugated with fluorescently antibodies to CD44, CD24 and ESA (stem cell markers), 7-AAD (viability marker) and Hoechst (Cell cycle). Cells were sorted into two populations CD44+/24lo/ESA+ (Stem-like cells) and CD44-/24hi/ESA- (Non stem-like cells) and according to cell cycle (Hoechst DNA profile) within the two populations into G0/1 and S/G2/M, and for comparison the whole cell population. Cell sorting experiments are explained within Supplementary Figure 2. The percentages of cells within each subpopulation sorted according to stem cell status (CD44-/CD24lo/ESA- vs CD44+/CD24hi/ESA+) and cell cycle phase (GO/1, S/G2M) are displayed within Supplementary Figure 3. Cells within the Stem-like cell population compared to the non stem-like cell population showed increased mammosphere population as expected and previously reported, example images are mammospheres are displayed within Fig. 2B. Separation of the stem-like cell population according to cell cycle, showed two very distinct populations within the currently defined stem-like breast cancer cells. Cells within G0/1 (non-proliferative) showed the greatest mammosphere formation compared to all other cell populations and in particular stem-like cells within the S and G2/M phase. These data highlight a heterogeneous population within the stem-like cells using currently defined cell surface markers, further demonstrate the relationship between stem-like cells and proliferation, and finally may have identified a purer population of stem-like cells with increased mammosphere activity.

### G0/1 Stem-like cells have increased migratory activity

**Figure 3:**
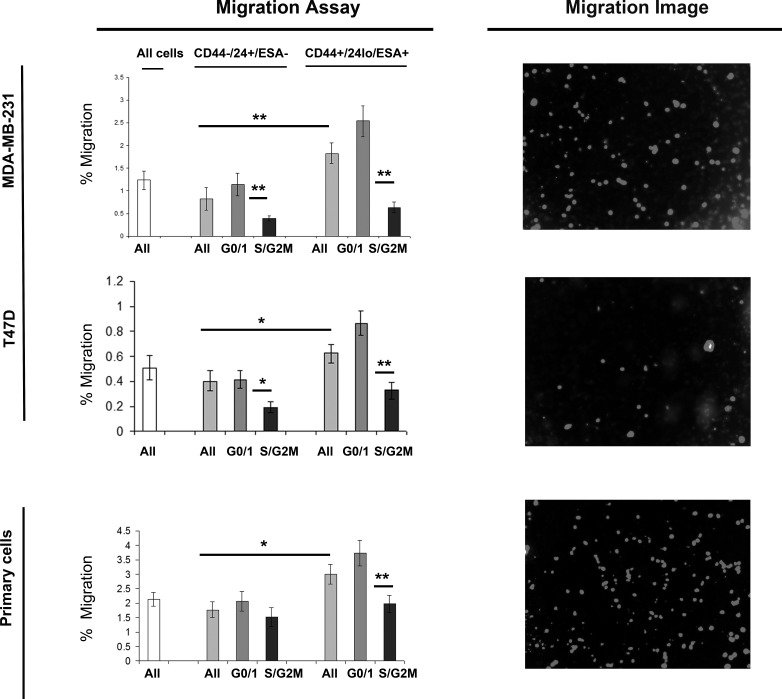
G0/1 Stem-like cells have increased migratory activity Breast cancer cell lines (MDA-MB-231 and MCF7) and primary human breast cancer cells were live cell sorted by FACS. Cells were sorted by ESA +/−, then CD44+/−, then CD24 lo/high to gain CD44+/24lo/ESA+ (Stem-like cells) and CD44-/24hi/ESA- (Non stem-like cells). These populations were sorted according to cell cycle (Heoest DNA profile) into G0/1 and S/G2/M, and for comparison the whole cell population (all). (A) transwell migration assayed. Bar charts represent the mean number of migrated cells/field of view, ±SEM. p values were generated using a two sided t-test assuming equal variance. * indicates significance, p<0.05 B) Representative images of DAPI positive cells following migration taken using a x40 objective.

We next evaluated further the relationship between cell cycle and migration using multi-colour live FACS cell sorting. (Fig. 3) Breast cancer cell lines (MDA-MB-231 and T47D) and primary human breast cancer cells were labelled as described above and sorted into two populations CD44+/24lo/ESA+ (Stem-like cells) and CD44-/24hi/ESA- (Non stem-like cells) and according to cell cycle (Hoechst DNA profile) within the two populations into G0/1 and S/G2/M, and for comparison the whole cell population. Cells within the Stem-like cell population compared to the non stem-like cell population showed increased migration. Separation of the stem-like cell population according to cell cycle, again showed two very distinct populations within the currently defined stem-like breast cancer cells. Cells within G0/1 (non-proliferative) showed the greatest migratory activity compared to all other cell populations and in particular stem-like cells within the S and G2/M phase.

### Stem cell signalling inhibitors specially inhibit G0/1 Stem-like cells

**Figure 4:**
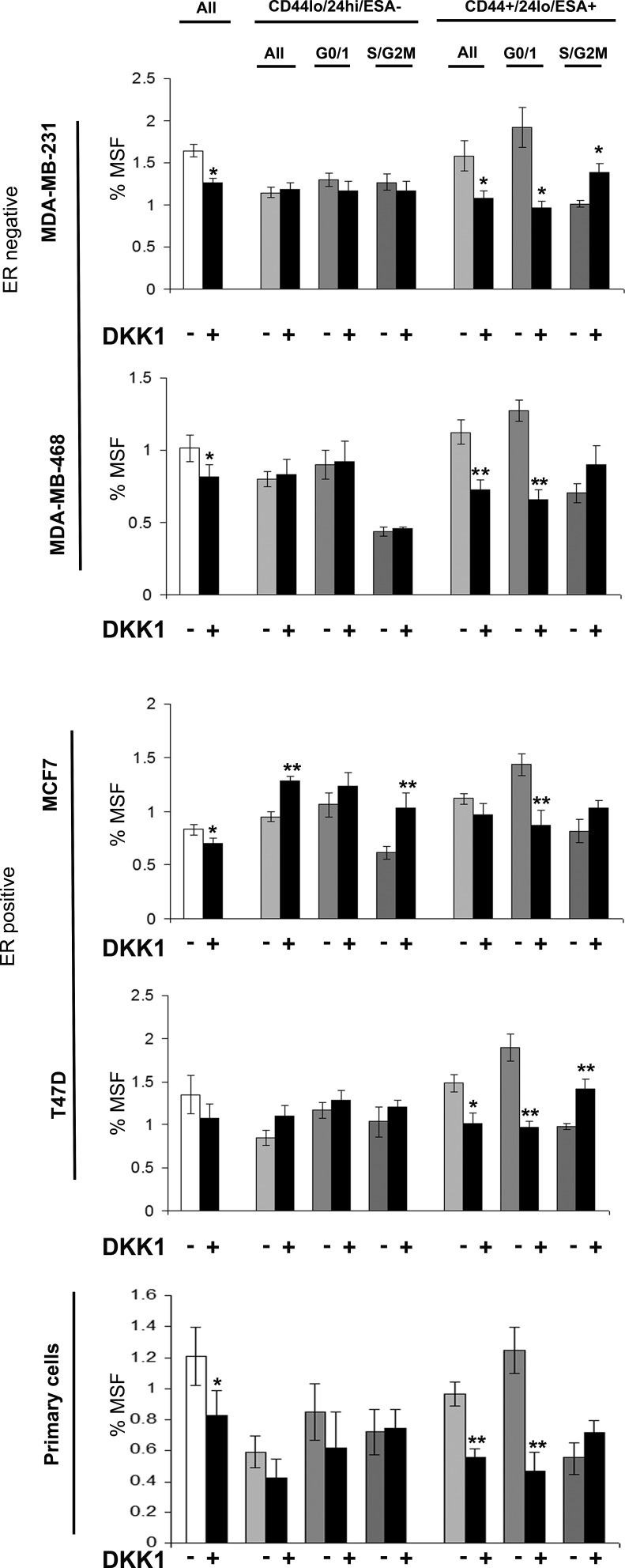
Wnt inhibitor DKK1 specifically inhibits G0/1 Stem-like cells Breast cell lines (MDA-MB-231, MDA-MB-468, MCF7 and T47D) and primary human breast cancer cells were live cell sorted by FACS. Cells were sorted by ESA +/−, then CD44+/−, then CD24 lo/high to gain CD44+/24lo/ESA+ (Stem-like cells) and CD44-/24hi/ESA- (Non stem-like cells). These populations were sorted according to cell cycle (Hoechst DNA profile) into G0/1 and S/G2/M, and for comparison the whole cell population (all). (A) mammosphere formation assayed +/− DKK1 (50ng/ml) (Black bars). Bar charts represent the % mammosphere formation, ±SEM. p values were generated using a two sided t-test assuming equal variance. * indicates significance, p<0.05

**Figure 5:**
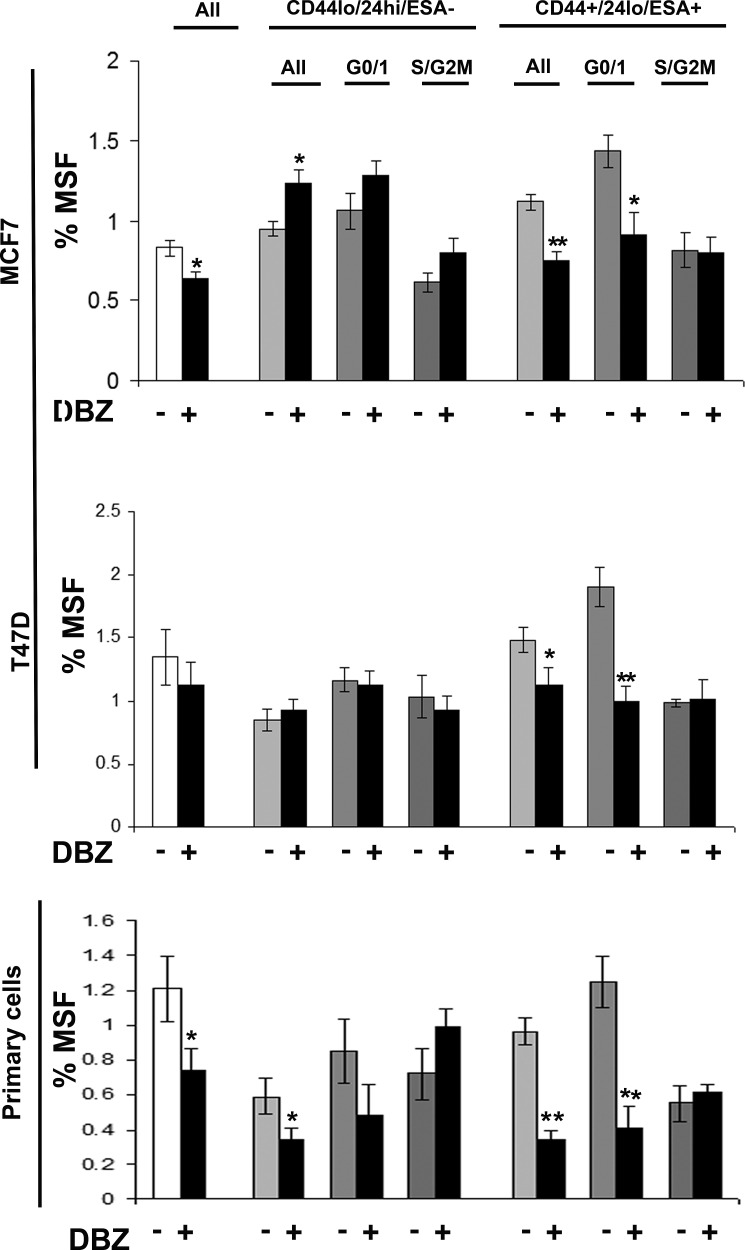
Notch inhibitor DBZ specifically inhibits G0/1 Stem-like cells Breast cell lines (MCF7and T47D) and primary human breast cancer cells were live cell sorted by FACS. Cells were sorted by ESA +/−, then CD44+/−, then CD24 lo/high to gain CD44+/24lo/ESA+ (Stem-like cells) and CD44-/24hi/ESA- (Non stem-like cells). These populations were sorted according to cell cycle (Heoest DNA profile) into G0/1 and S/G2/M, and for comparison the whole cell population (all). (A) mammosphere formation assayed +/− DBZ (10μM) (Black bars). Bar charts represent the % mammosphere formation, ±SEM. p values were generated using a two sided t-test assuming equal variance. * indicates significance, p<0.05

Breast cancer cell lines (MDA-MB-231, MDA-MB-468, MCF7 and T47D) and primary human breast cancer cells were labelled as described above and sorted into two populations CD44+/24lo/ESA+ (Stem-like cells) and CD44-/24hi/ESA- (Non stem-like cells) and according to cell cycle (Hoechst DNA profile) within the two populations into G0/1 and S/G2/M, and for comparison the whole cell population. Mammospheres were plated and treated with inhibitors of stem cell signalling, DKK1 (Fig 4) to inhibit Wnt signalling and DBZ (Fig 5) to inhibit Notch signalling. DKK1 and DBZ inhibited mammosphere formation when tested in whole cell populations, and within the stem-like cell populations. What is most notable is the highly significant decrease in mammospheres observed following treatment of G0/1 stem-like cells, accompanied by an increase in mammosphere formation within S/G2M cells. These data again provide evidence for two distinct populations of cells within the currently defined stem-like cell.

### Stem cell signalling inhibitors decrease self renewal of MCF7 stem-like cells

**Figure 6:**
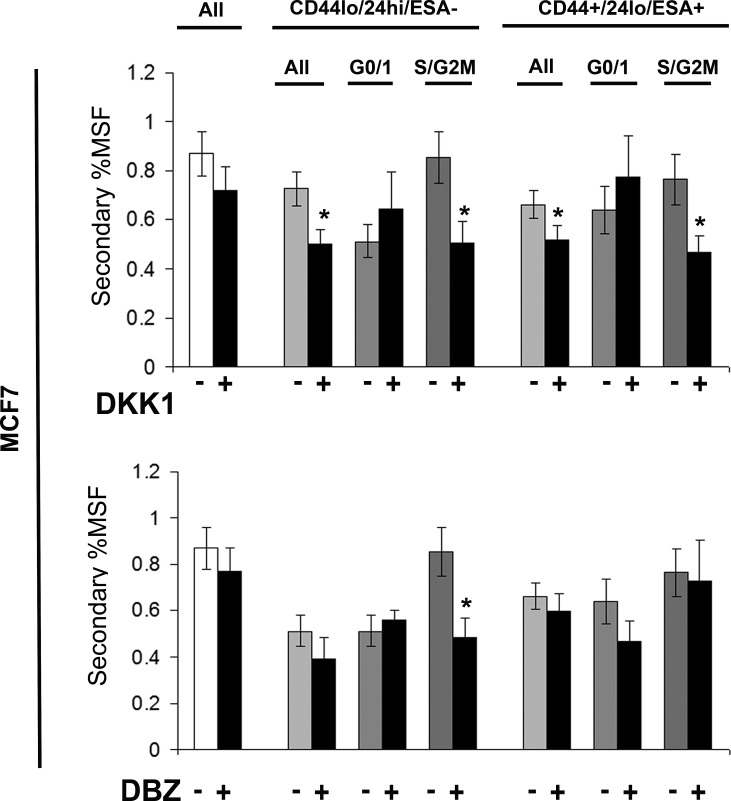
Stem cell signalling inhibitors decrease self renewal of MCF7 stem-like cells Breast cell line (MCF7) were live cell sorted by FACS. Cells were sorted by ESA +/−, then CD44+/−, then CD24 lo/high to gain CD44+/24lo/ESA+ (Stem-like cells) and CD44-/24hi/ESA- (Non stem-like cells). These populations were sorted according to cell cycle (Hoechst DNA profile) into G0/1 and S/G2/M, and for comparison the whole cell population (all) and mammosphere formation assayed +/− DKK1 (50ng/ml) or +/− DBZ (10 μM) (Black bars). After five days of growth MS were collected and secondary MS plated, and counted after a further 5 days. Bar charts represent the % secondary mammosphere formation; ±SEM. p values were generated using a two sided t-test assuming equal variance. * indicates significance, p<0.05

Breast cancer cell line (MCF7) was labelled as described above and sorted into two populations CD44+/24lo/ESA+ (Stem-like cells) and CD44-/24hi/ESA- (Non stem-like cells) and according to cell cycle (Hoechst DNA profile) within the two populations into G0/1 and S/G2/M, and for comparison the whole cell population. Mammospheres were plated and treated with inhibitors of stem cell signalling, DKK1 (Fig 6) to inhibit Wnt signalling and DBZ (Fig 6), after five days mammospheres were counted and re-plated, and secondary mammosphere formation calculated, and indicated of self-renewal. DKK1 and DBZ treatment showed a small decrease in secondary mammosphere formation, with DKK1 having the greatest effects on secondary mammosphere formation. DKK1 decreased the secondary mammosphere formation of stem-like cells; however where DKK1 increased the primary mammosphere formation of stem-like cells in S/G2M, the secondary mammosphere formation was greatly reduced. These data suggest that DKK1 treatment decreases primary mammosphere formation, of stem-like cells and their subsequent self renewal. However in stem-like cells within S/G2M, DKK1 treatment increased primary mammosphere, with a reduction in secondary mammosphere formation suggesting that DKK1 increases proliferation of this population, but not self-renewal.

## DISCUSSION

Clinical cancer research has focused much effort on inhibition of cellular proliferation, with the aim of reducing tumour growth and size, and in the long term increasing patient survival. This strategy and the use of such drugs as Paclitaxol target proliferating cells, and can result in decreased tumour growth and survival [14]. Despite the advances in treatments and their effectiveness in de-bulking the tumour, many patients are either unresponsive or eventually show local or distant recurrence. Certain therapies have been demonstrated to leave unharmed a population of stem-like cells, marked by anoikis resistance (pre-cursor to mammosphere) and the expression of stem cell markers CD44+/24lo [12, 13].

We have identified a clear inverse correlation between proliferation and migration/mammosphere formation (stem-like cell activity) using four breast cancer cell lines and primary human breast cancer samples. We used live cell sorting to separate cell populations according to proliferation status, for assessment of migration and mammosphere formation activity. Cells within G0/1 cell cycle phase showed consistent increased migration and despite having low proliferative capability, showed increased mammosphere formation. Stem cells with low proliferative capacity need only undergo one act of self-renewal in order to produce progeny capable of proliferating and forming the major mass of a mammosphere. Whereas, non stem cells with high proliferative capability lack the ability to survive in anoikis, self-renew and ultimately lack the ability to form mammospheres.

These data suggest that low-proliferative cells, which would escape anti-proliferative treatment, are increased in stem cell-like activity, marked by mammosphere formation, and migration. Un-successful targeting of these non-proliferative cells could leave an enriched population of high migratory cells with stem cell-like activity, likely to decrease survival through the development of metastasis and drug resistance.

Live cell sorting using a combination of stem cell markers and cell cycle analysis, allowed further interrogation of the currently defined stem-like cells (CD44+/CD24lo/ESA+) and assessment of the relationship between proliferation, migration and stem cells. Firstly we demonstrated that stem-like cells (CD44+/CD24lo/ESA+) were more migratory in both breast cancer cell lines and primary breast cancer samples, consistent with published research [15-17]. Secondly, we showed that within the currently defined stem-like cells exist two distinct populations of cells determined by their cell cycle status. G0/1 CD44+/CD24lo/ESA+ cells showed the greatest migratory and mammosphere activity, suggesting that this sub-population of low proliferative cells maybe more stem-like cell, perhaps a true “stem cell”, whilst cells within S/G2M may display a more progenitor-like phenotype with increased ability to proliferate.

The inclusion of cell cycle analysis in addition to current markers may improve future isolation and analysis and stem cells in breast cancer and may be applicable to other cancer types. To add further to our evidence of two distinct populations of cells within the CD44+/CD24lo/ESA+ populations we treated cells with know stem cell inhibitors of the Wnt and Notch pathway. Addition of Wnt and Notch signalling inhibitors reduced the mammosphere formation of breast cancer whole cell populations as previously reported [18-20]. However, stem cell pathway inhibition had opposing effects on CD44+/CD24lo/ESA+ dependant upon cell cycle, with decreased mammosphere formation in G0/1 cells and an increase in S/G2M. Subsequent secondary passage of mammospheres was reduced suggesting that DKK1/DBZ treatment did not increase self-renewal of S/G2M cells however self renewal was decreased for stem-like cells.

In conclusion, these data show the co-ordination and relationship between migration, stem cells and proliferation, and support our hypothesis that stem-like cells can be divided according to proliferation status, to further enrich the stem-like population and generate a purer more homogenous representation of the potential true stem cell population. Furthermore, clinical treatments directed to inhibiting stem cell signalling pathways used in conjunction with current methods may be an essential part of breast cancer therapy to overcome current drug resistance and increase long term survival.

## MATERIALS AND METHODS

### Patient Samples

Samples were collected with informed consent from three breast cancer patients including both primary and metastatic cells. Primary solid tumour samples were dissected into 1 mm pieces and incubated at 37°C for 16 hours in 1x Collagenase/Hyaluronidase mixture (STEMCELL Technologies Inc., #07912 ) in DMEM:F12/15mM HEPES (Sigma-Aldrich Co., #D6421). Pleural effusion and ascites samples were collected from patients with metastatic breast cancer during standard drain protocol. Following extraction from tissue (as detailed above) or collection of metastatic fluid, cells were collected by centrifugation at 800g and resuspended in PBS. Blood cells were removed by centrifugation through 0.5 volumes of Lymphoprep solution (Axis-Shield plc, #1114544) at 600g. Cells were cultured in DMEM:F12/20% FCS/0.1% non-essential amino acid solution/2.5mM L-glutamine/PenStrep (P/S).

### Ethics Statement

Any experimental research reported in the manuscript has been performed with the approval of an appropriate ethics committee and in compliance with the Helsinki Declaration. All samples were collected with informed written consent. Patient derived metastatic breast cancer cells were collected with approval from Tameside and Glossop Local Research Ethics Committee (COREC # 05/Q1402/25). Primary solid tumour samples were obtained from the Manchester Cancer Research Centre (MCRC) Biobank, UK (project ID: 09_GOLA_02). The role of the MCRC Biobank is to distribute samples and therefore, cannot endorse studies performed or the interpretation of results.

### Cell Lines

All cell lines were purchased from ATCC; MDA-MB-231, MDA-MB-468 (ER negative) MCF7, and T47D (ER positive). Cell lines were authenticated by multiplex PCR assay using the AmpFlSTR Indentifiler PCR amplification kit (Applied Biosystems, Life Technologies Corporation, #4322288) and confirmed as mycoplasma free. MDA-MB-231 and MDA-MB-468 were cultured in RPMI complete medium (RPMI/10% FCS/1% Sodium pyruvate/2mM L-glutamine/PenStrep) whilst MCF7 and T47D were grown in DMEM complete medium (DMEM/10% FCS/2mM L-glutamine/PenStrep). Cells were maintained in a humidified incubator at 37^o^C at an atmospheric pressure of 5% (v/v) carbon dioxide/air.

### Flow Cytometric Sorting

Cells were resuspended at ≤1 × 10^6^ in 100 μL containing 10μg/ml Hoechst 33342 (Sigma) and incubated at 37°C for 45 minutes. For experiments to separate G0 and G1 populations, cells were additionally labelled with Pyronin Y (Sigma). Cells were resuspended in sorting buffer (PBS containing 0.5% bovine serum albumin, 2 mmol/L EDTA) and 7-AAD added as a marker of viability (5μl per 1×10^6^ cells). Cells were then sorted according to cell cycle profile (G0/1, S and G2M) based on their fluorescence using the BD influx. In addition, following Hoechst 33342 labelling, cells were incubated with pre--conjugated primary antibodies BEREP4-FITC (1:10; Dako), CD44-APC (1:20; BD Pharmingen), and CD24-PE (1:10; Beckman Coulter) for 10 min at 4°C. The cells were washed in PBS and centrifuged at 800 × g for 2 min and resuspended in sorting buffer, with the addition of 7-AAD. Cells were then sorted firstly into CD44+ /24low / ESA+ (“Stem cells”) and CD44-/24hi /ESA- (“Non-stem cells”). The CD24low cell population gated by FACS was the lowest quintile of CD24-positive cells plus all the CD24-negative cells. Additional sorting was then applied to these two populations based upon the cell cycle profile produced by Hoechst 33342 fluorescence, into G0/1 and S/G2M populations.

### Mammosphere Culture

A single cell suspension was prepared using enzymatic (1x Trypsin-EDTA, Sigma Aldrich, #T3924), and manual disaggregation (25 gauge needle). Patient-derived breast cells did not require the addition of trypsin, a single cell suspension was created using manual disaggregation. Cells were plated at a density of 500 cells/cm^2^ in mammosphere medium (DMEM-F12/B27/20ng/ml EGF/PenStrep) in non-adherent conditions, in culture flasks coated with (2-hydroxyethylmethacrylate) (poly-HEMA [Sigma]). Cells were grown for 5 days and maintained in a humidified incubator at 37°C at an atmospheric pressure in 5% (v/v) carbon dioxide/air. Spheres >50μm were counted using an eye piece graticule, and the percentage of cells plated which formed spheres was calculated and is referred to as the percentage mammosphere formation (%MSF).: Live cell imaging of mammospheres was performed using an Olympus CKX41 inverted light microscope at x40 magnification, scale bar represents 50μm. Secondary Mammospheres: After 5days of culture, mammospheres were collected by centrifugation at 800rpm for 2 minutes, dissociated by manual disaggregation into a single cell suspension at re-plated at 500 cells/cm^2^ in mammosphere medium for a further 5 days. Culture of cells in these conditions allows the survival of stem-like cells and subsequent spheroid growth from a single cell using both cell lines and primary tissue samples. Mammospheres grow at a similar rate when plated as single cells or at higher densities indicating that mammospheres are truly clonal structures and not formed through aggregation [21, 22].

### Migration Assay

Transwell chambers with a diameter of 6.5 mm and a pore size of 8 μm were used to assess migration (Corning, Inc. #3422). Migration chambers were incubated with 150 μl serum-free media for an initial equilibrium period of 1 hour. Cells (transfected the day before) were resuspended in serum-free media and 50 000 cells were added to each migration chamber. Next, chambers were placed into wells containing 600 μl 10% FCS medium, and cells were allowed to migrate for 5 hours (MDA-MB-231, MDA-MB-468) or overnight (MCF7, T47D and primary breast cells). Cells that had not migrated were removed with a cotton swab, whereas the migrated cells situated on the lower side of membranes were fixed in 4% paraformaldehyde for 25 minutes. Membranes with the migrated cells were mounted on glass slides for DAPI staining and counted using a fluorescent microscope (cells in six X10 magnification fields were counted). Experiments were repeated at least three times.

### Inhibitors of stem cell signalling

Wnt pathway inhibition. MDA-MB-231, MDA-MB-468, MCF7, and T47D cells and primary human breast cancer cells were plated into MS culture following FACS cell sorting and treated with a single dose of human recombinant Dickkopf 1( DKK1) (R and D systems) (50ng/ml).

Notch pathway inhibition. MCF7, T47D cells and primary human breast cancer cells were plated into MS culture following FACS cell sorting and treated with a single dose of Dibenzazepine (DBZ; 10μM).

### Statistical Methods

Throughout the paper data is represented as mean ±SEM taken over a minimum of three independent experiments. Statistical significance was measured using parametric testing, assuming equal variance, with standard t-Tests for two paired samples used to assess difference between test and control samples.

## SUPPLEMENTARY MATERIAL FIGURES


