# Supplementary material for: Co-ordination of cell cycle, migration and stem cell-like activity in breast cancer
Source: Oncotarget. 2014 Aug 4;5(17):7833–42. doi: 10.18632/oncotarget.2312 (PMC4202164; doi:10.18632/oncotarget.2312)
Supplement: Supplementary file 1 [file oncotarget-05-7833-s001.pdf]

Co-ordination of cell cycle, migration and stem cell-like activity in breast cancer

Supplementary Material

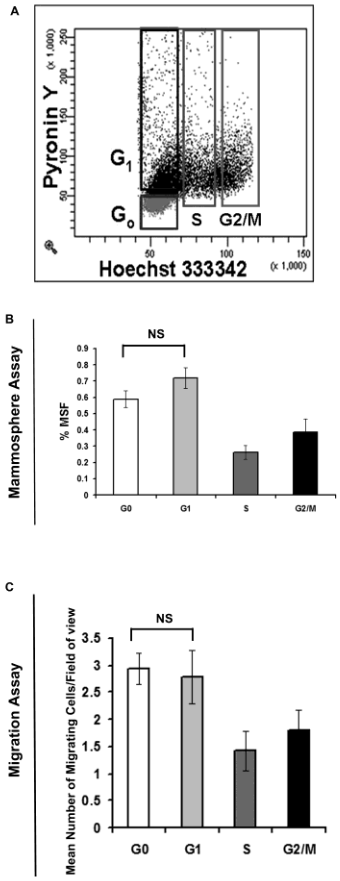

Supplementary Figure 1: Pyronin Y and Hoescht 33342 co-labelling show no difference between G0 and G1 cells. Breast cell line MDA-MB-231 cells were live cell sorted by FACS according to Hoechst and Pyronin Y staining to distinguish between G0, G1, S, G2/M populations. (A) FACS plot of Hoechst 33342 and Pyronin Y gating strategy used to perform live cell sorting. (B) Mammosphere formation and (C) transwell migration assayed. Bar charts represent the mean number of migrated cells/field of view and % mammosphere formation, ±SEM. p values were generated using a two sided t-test assuming equal variance. No significant (NS) differences were observed between G0 and G1 cell populations.

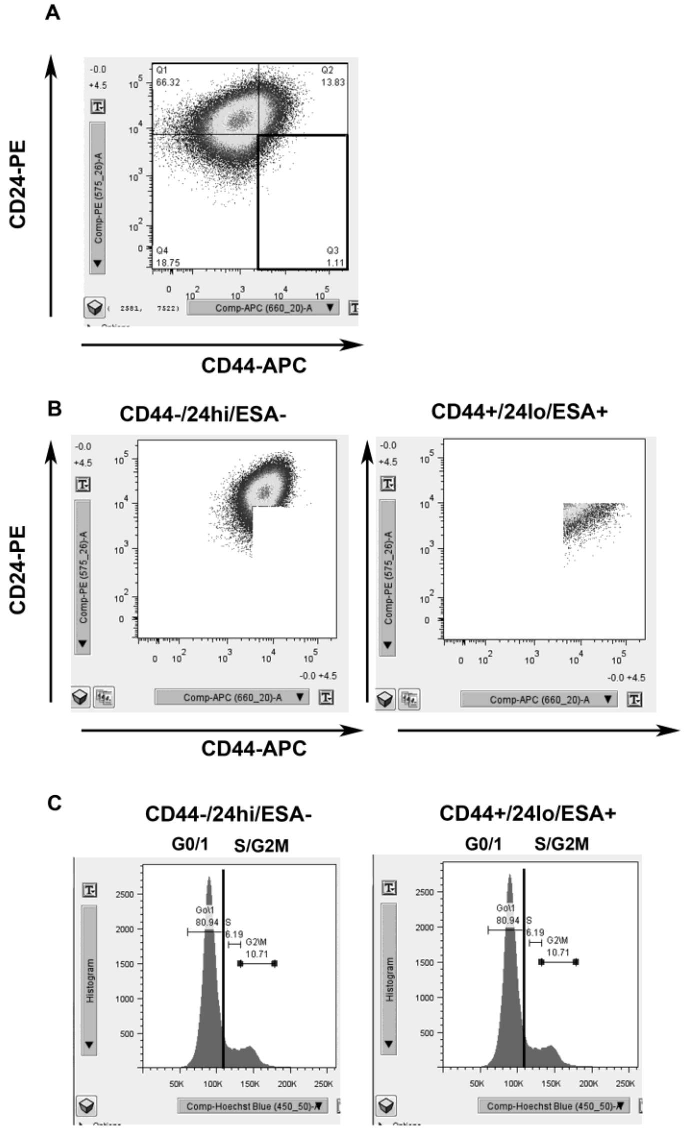

Supplementary Figure 2: Diagrammatic representation of live cell sorting A) FACs plot of CD24-PE and CD44-FITC expression in whole cell populations. B) FACS plot of CD24PE and CD44-FITC showing gating used to perform live cell sorting into CD44-/CD24hi/ESA- (left panel) and CD44+/CD24lo/ESA+ (right panel) populations, ESA positive and negative gates were applied separately. C) These two populations were further sorted according to cell cycle. FACs plots show histograms of Hoechst 33324 fluorescence used to determine cell cycle, separating G0/1 from S/G2M.

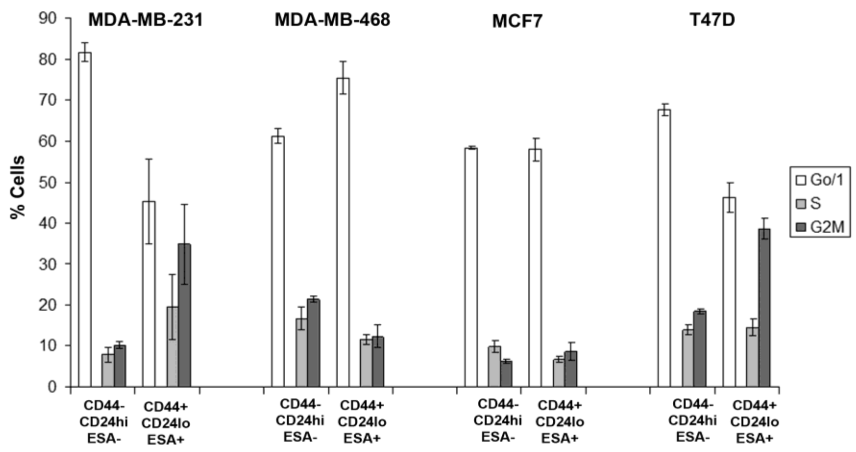

Supplementary Figure 3: Cell percentages within subpopulation of FACs sorted breast cancer cell lines. Breast cancer cell lines (MDA-MB-231, MDA-MB-468, MCF7 and T47D) were analysed by FACS. Cells were separated into CD44+/24lo/ESA+ (Stem-like cells) and CD44-/24hi/ESA- (Non stem-like cells), and the number of cells in each cell cycle phase (G0/1 and S/G2/M) calculated, as determined by Hoechst DNA profiles. Bar chart represents the % cells within each sub-population ±SEM.
